# Cadmium: A Focus on the Brown Crab (*Cancer pagurus*) Industry and Potential Human Health Risks

**DOI:** 10.3390/toxics10100591

**Published:** 2022-10-06

**Authors:** Ronan Lordan, Ioannis Zabetakis

**Affiliations:** 1 Department of Biological Sciences, University of Limerick, V94 T9PX Limerick, Ireland; 2 Health Research Institute, University of Limerick, V94 T9PX Limerick, Ireland; 3 Bernal Institute, University of Limerick, V94 T9PX Limerick, Ireland; 4 Institute for Translational Medicine and Therapeutics, Perelman School of Medicine, University of Pennsylvania, Philadelphia, PA 19104, USA

**Keywords:** cadmium, crab, crustaceans, heavy metal toxicity, nutrition, pollution, biomagnification, biomonitoring

## Abstract

Cadmium is a major health risk globally and is usually associated with pollution and anthropogenic activity. The presence of cadmium in food is monitored to ensure that the health and safety of consumers are maintained. Cadmium is ubiquitous in the Asian and Western diets, with the highest levels present in grains, leafy greens, and shellfish. As part of their natural lifecycle of moulting and shell renewal, all crustaceans—including the brown crab (*Cancer pagurus*)—bioaccumulate cadmium from their environment in their hepatopancreas. The brown crab is an important species to the crab-fishing industries of many European countries, including Ireland. However, the industry has come under scrutiny in Europe due to the presence of cadmium in the brown crab meat intended for live export to Asia. This review explores evidence regarding the effects of cadmium consumption on human health, with a focus on the brown crab. Differences in cadmium surveillance have given rise to issues in the crab industry, with economic consequences for multiple countries. Currently, evidence suggests that brown crab consumption is safe for humans in moderation, but individuals who consume diets characterised by high levels of cadmium from multiple food groups should be mindful of their dietary choices.

## 1. Introduction

**Figure 1:**
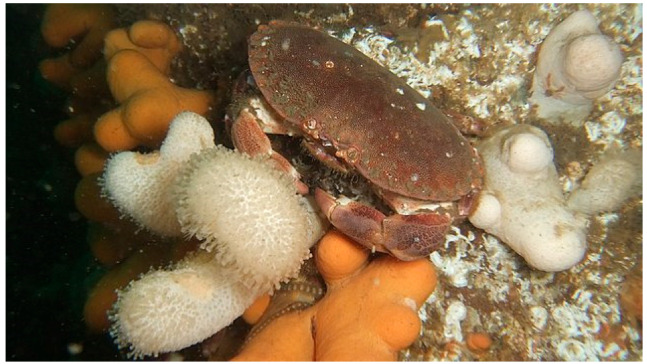
The brown crab (*Cancer pagurus*). Reproduced with permission from [23], licensed under CC BY 2.0.

Cadmium is a major health risk globally and is associated with pollution and anthropogenic activity. Consequently, cadmium levels in foods are monitored to ensure the health and safety of consumers [1]. Some foods are more likely to contain cadmium than others. These foods include rice, potatoes, leafy greens such as spinach, and various seafoods such as crustaceans (e.g., lobster, prawns, and crab) [2,3]. Crab fishing is an important industry around the world, including in Ireland and Northern Europe, where the brown crab (*Cancer pagurus*) (Figure 1) is the main species traded. The brown crab is of considerable value to the European economy, contributing to the income of the communities around the coastlines of trading nations such as Ireland [4,5]. The brown crab is caught off the Irish, Atlantic, and Mediterranean shores and occasionally found inshore in countries such as Norway using baited traps called pots or creels, which can be set individually or in strings [6].

The crab-fishing industry in general has come under scrutiny for cadmium levels present in the meat of frozen products and live exports to Asia [7,8,9]. Cadmium and other heavy metals bioaccumulate in the hepatopancreas of crustaceans, including the brown crab, due to this organ’s detoxifying function [10,11,12,13,14]. Cadmium may also be present transiently in low concentrations in the haemolymph [15].

Strict regulations exist in some Asian countries, such as the People’s Republic of China (PRC) and Hong Kong, where regulatory authorities require testing of cadmium levels of the combined white and brown meat of all crabs imported from abroad [16]. Ensuring the health of consumers is of critical importance; hence, the strict regulation of food products. However, the methods employed to sample and monitor cadmium levels in the crab industry generally appear to vary [17], as discussed in this review. In the European Union, cadmium limits are prescribed for the white crab meat intended for consumption (0.5 mg/kg) [18,19,20], whereas the same limits are applied to the total of the white and brown crab meat in the PRC and Hong Kong (0.5 mg/kg) [9,17,21]. These discrepancies have caused considerable tension between these trading nations [7]. However, cadmium is an issue for all crab-producing nations, but not all nations have the same issues with exports to Asian countries; therefore, it is possible that the “political climate between trading countries” may play a role [9], with preferences evident for some countries over others [22].

Chronic consumption of cadmium due to a cumulative dietary intake from cadmium-exposed foods is dangerous and carries a serious risk of toxicity [24]. Although cadmium toxicity is extremely rare in modern times [25], monitoring cadmium levels in various foods is nevertheless a critical safety net to maintain health.

Brown crab is mostly treated as a delicacy and, thus, is consumed in low amounts in most countries. However, there are countries that are exceptions, including South Korea [26], Portugal [27], and Norway [28], where regular consumption is common among some of their populations. For the consumption of any species of crab, factors such as the age of the crab, the part of the crab consumed, and the origin of the crab may contribute to the ingestion of cadmium over time [29,30,31]. Consuming the white meat of the crab from the appendages, claws, and legs, and avoiding the cephalothorax that contains the gonadal tissue and hepatopancreas (known as the brown meat or tomalley), can be considered low-risk, as cadmium is mostly concentrated in the latter tissues [27]. While the brown meat is edible and enjoyed around the world due to its distinctive flavour, as a precaution, some health authorities and those in the scientific domain advise against its regular consumption from crab [9,32,33] and other crustaceans [34]. In 2009, a scientific opinion was published followed by an information note in 2011 by the European Commission, who recommended that member states should advise consumers about the consumption of brown crab meat due to the higher levels of cadmium in the cephalothorax [35]. However, as discussed in this review, a person’s diet as a whole must be taken into consideration when attempting to determine their intake of cadmium. Thus, we consider the consumption of crab in relation to various dietary patterns, the monitoring of cadmium in crabs, and human studies of brown crab consumption.

## 2. Brown Crab (*Cancer pagurus*)

**Figure 2:**
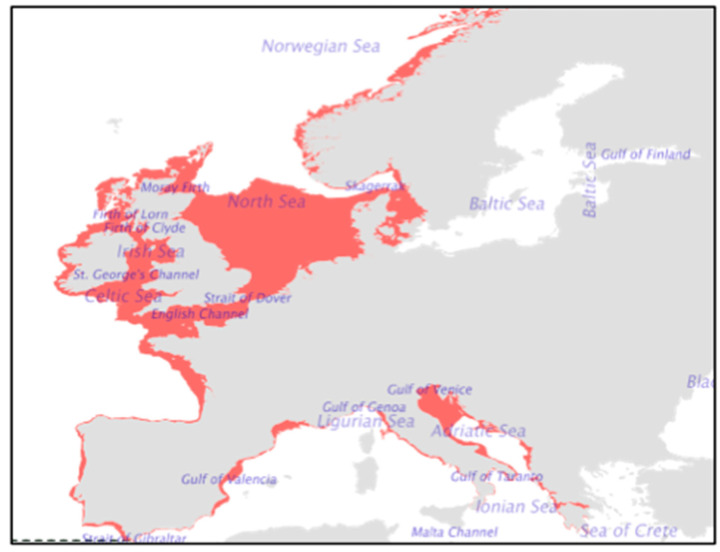
A diagram of the geographic distribution of brown crabs in Europe. Reproduced with permission from the European Market Observatory for Fisheries and Aquaculture Products [9].

The brown crab is a long-lived, benthic, carnivorous, nocturnal predator species that resides on the seafloor, commonly found at depths of 6–100 m, but they are generally found between 6 and 40 m, where smaller crabs can be seen closer to the intertidal zone. The male brown crab is easily identified by its large, black-tipped claws and its “pie-crust” edged carapace (Figure 1), while the female brown crab has a domed and rounded carapace, which offers greater meat yield. However, the most reliable and common method used for sex determination is to inspect the shape of their tail, where the male abdomen is relatively narrow, while that of the females is wider [36,37,38]. Brown crabs largely consume molluscs and other decapod species. They achieve a carapace width of up to 270 mm in males and 250 mm in females, although there is regional variation and there have been reports of larger males reaching 300 mm. They are 5 years old when they reach minimum landing size, but they can live to 25–30 years, with some living to almost 100 years old. Brown crab can moult (the process of ecdysis) several times per year when young but less often as they become larger in size, even up to once every 4 years [4,31,37,39,40]. This can make it difficult to determine the age of a crab. After moulting, mating occurs generally from July to September when the female carapace is soft. The male transfers spermatozoa to the female, who stores the spermatozoa in a specialised organ (the spermatheca) by forming a plug, pending internal fertilisation, which usually occurs 1–14 months post-mating. The female spawns the fertilised eggs onto the pleopods and carries them over winter into the hatching season from spring to summer. During this time, the (berried) females generally remain in pits dug into the sediment or under rocks, where they mostly refrain from movement or feeding. Notably, females can spawn and inseminate the eggs without any need for mating up to several times, due to the presence of sperm plugs that can keep the spermatozoa viable for up to 3 years. Hatching can occur at any time in this period depending on numerous environmental conditions, including water temperatures and latitude. As many as 1–4 million eggs may hatch from one female brown crab, indicating their high fecundity. Because crabs are known to be migratory due to mating patterns, the hatchlings may be found over a vast distance [4,37,39,41,42,43], although the distribution and density of brown crabs are affected by many factors—not only fecundity or migration. For example, water temperature and access to food are additional important factors, and there tends to be greater population density inshore [37]. The biology of brown crabs is important to consider in order to understand their distribution and their availability for human consumption. The geographic distribution of brown crabs in Europe is presented in Figure 2.

## 3. The Importance of the Brown Crab Fishing Industry and the Challenge of Cadmium: Ireland as an Example of a Crab-Trading Nation

The seafood industry is significant to the Irish economy and contributes to a vibrant export trade with European and Asian countries, especially the PRC. The brown crab, or “portán dearg”, is the heaviest Irish crab and is landed by most major and some minor fishing ports in the country. The brown crab is a non-quota species and can be legally caught by vessels operating with a polyvalent or potting license [44]. Globally, the brown crab is a seafood species with increasing value, totalling a catch greater than 50,000 tonnes per year [45]. Exports of crustaceans and molluscs to the PRC from Ireland in 2018 increased by 68%, accounting for a total of 12,700 tonnes of seafood and growing the market to almost EUR 46 million [46]. Ireland has become one of the top three producers of brown crab products in Europe [47]. In 2018, landings of Irish brown crab reached 5500 tonnes, representing EUR 1 million in domestic sales and EUR 60 million in exports. Additionally, the price of Irish brown crab that year increased by 58% due to considerable demand from the Asian market, making brown crab the third most valuable export species that year. This increase in exports and price is of particular value to small-scale inshore fishermen in coastal communities around Ireland [48], although it should be noted that the Irish market was affected by the coronavirus disease 2019 (COVID-19) pandemic, with lower exports reported in 2020. The Irish brown crab exports reached a total of 2878 tonnes in 2020, representing a decrease of 47% compared with 2019, recording an exports value decrease of 27% for the same period [9].

Chinese food import authorities have always been concerned regarding the levels of cadmium in all crustacean species originating from multiple countries, as they may exceed their limits of 0.5 mg/kg [9,49,50]. Ireland is not the only nation affected, as there are also reports of similar issues in Britain, Spain, France, Norway, Portugal, the Netherlands (mitten crab), the United States (Dungeness crab), and even Taiwan for various crab species exported to the PRC [8,9,49,51,52,53]. The main European countries exporting brown crab to the PRC have traditionally been the United Kingdom, Ireland, and France [6]. Initially, those countries used the basic health certificates (HCs) approved by their individual regulatory bodies, which were relatively non-specific regarding heavy metal testing but guaranteed that seafood was not contaminated by pollutants [9,54].

Over the past decade, the supply of brown crab from the main European exporting countries has been rejected due to cadmium for a period one or more years, and with high-level negotiations ongoing including delegations from the PRC visiting the exporting countries [55,56,57]. In all cases, more specific testing and health certificate (HC) formats have been agreed, which has restored some level of export of brown crab to the PRC. In Ireland, the Sea Fisheries Protection Authority (SFPA) is responsible for the issuance of Irish HCs and operates a monitoring plan whereby samples are collected and tested in a state laboratory [9,58]; however, difficulties still persist, particularly around the export of live crabs under these systems.

Questions have been raised in relation to whether the strict limits of cadmium intake in seafood might be misguided. Indeed, in the PRC there has been public consultation to raise the permitted level of cadmium in brown crab from 0.5 mg/kg to 3.0 mg/kg, but an update on this consultation has not been released yet. It is undeniable that cadmium does indeed accumulate in humans due to dietary exposure and that this does have deleterious effects on health, as discussed later in the review. However, one must consider the entirety of the dietary intake of cadmium from various sources before limits of cadmium in a specific food are imposed for specific populations. Equally, the frequency of consumption of a particular food product that is naturally high in cadmium must also be considered when determining dietary guidance on cadmium-containing products. This concept is discussed further in Section 5.

## 4. Cadmium Accumulation and Monitoring in Crabs and Crustaceans

The Industrial Revolution over the last two centuries has led to anthropogenic-derived pollution that ultimately has negatively affected various global biomes and ecosystems. While cadmium is ubiquitous in the environment at very low levels, industrial and technological advancement has occurred at the expense of the environment, with an increased release of cadmium (Cd^2+^) into the environment. Cadmium is an element that can accumulate in the food chain, with potential human and ecosystem risks [59,60]. Cadmium can bioaccumulate in various marine organisms, but most prominently within crustaceans such as lobsters, crabs, crayfish, and prawns. The elemental composition of crabs has mainly been studied in crabs from the Norwegian and Scottish coasts and the English Channel [31]. See Table 1 for an overview of cadmium measurements in brown crabs. As mentioned above, crabs and other crustaceans accumulate metals mostly via diet, with some negligible amounts coming from their environment [61,62,63].

Several methods have been employed over the years to detect cadmium in biological samples. The most effective method to date that is commonly used is inductively coupled plasma mass spectrometry (ICP-MS), where cadmium can be detected at levels as low as 0.003 µg/L [67,68]. Indeed, most food and environment surveillance agencies use ICP-MS or similar methods to monitor cadmium in crabs [17,69]. However, alternative methods are available and have been used to measure cadmium in crabs, as shown in Table 1. For example, atomic absorption spectroscopy (AAS) is another effective analytical tool used to measure cadmium in biological materials [68]. Graphite furnace atomic absorption spectroscopy (GFAAS) was previously a common method used for the detection of cadmium in foods, with a sample detection limit of 0.4 µg/L [70]. However, other methods of cadmium detection exist that are not commonly used today for seafood analysis. For instance, radiochemical neutron activation analysis (RNAA) is a method that has been used to detect cadmium burden in humans, but its detection limits are not preferential [68]. Other methods include differential pulse ASV [71] and the calorimetric dithizone method [68].

Various factors affect cadmium accumulation in crab species. In *Carcinus maenas* (shore crab), factors such as ovarian maturation, moulting stage, condition (e.g., water content of the crab), tissue hydration, sex, and size all affect cadmium bioaccumulation [62,63,72,73,74]. In brown crabs specifically, there is evidence that cadmium levels differ due to location and cooking (which increases cadmium in claw meat while reducing the concentration in the inner meat), and there is a correlation between crab size and levels of cadmium in the hepatopancreas [30,75], which implies that cadmium accumulates as the crab ages. However, season, moulting, and gonad maturation have limited effects on cadmium concentrations in brown crabs [31]. Although no account has been provided regarding the age of the crabs sampled and the correlation between age and cadmium levels, this is likely because crustaceans are very difficult to age due to the process of moulting their exoskeleton throughout their life and their indeterminate growth [76]. Processing conditions are also an important consideration. Frozen crab that has been defrosted may leech cadmium with the haemolymph, which may be lost during the cooking process. Indeed, claws taken from frozen crab before thawing had lower cadmium levels than claws taken from the carapace after thawing, indicating that there can be redistribution of cadmium in the crab. This may indicate biases when assessing cadmium levels in crabs that have been processed [30]. Studies have also linked cadmium levels to seasons, where lower levels have been detected in the summer months [63,72], potentially due to the shorter biological half-life of cadmium as the temperature increases during summer [63]. However, the effect of season was inconclusive in another study [31].

The tissue hydration and water content of the crab have significant effects on the levels of cadmium measured in the crab—particularly when considering that a lot of trade involves live transport of crabs. This has significant implications for obtaining accurate measurements of cadmium in crabs, as once taken from its environment the animal will begin to lose water content. For live export, it is important that the crabs are in a suitable environment as they are osmoconformers and, therefore, are reliant on their external environment to maintain body fluid osmolarity [77]. The longer the crab is out of the water before testing and consumption, the more likely the cadmium is to concentrate. However, it should be noted that only some water loss in the interior organs can be tolerated before the crab dies and is no longer suitable for human consumption. Indeed, feeding is also an important consideration for export, as the hepatopancreas and the reproductive tissue may fill the crab’s entire body or may dwindle during periods of poor feeding [78]. These changes in biological condition can, in turn, affect the overall relative levels of cadmium in laboratory samples during testing. Therefore, there are potentially discrepancies in testing for cadmium, as various laboratories will have different schedules for testing and follow different butchering, sample preparation, and testing protocols, and crab samples may be in storage for a considerable amount of time prior to testing. This is an important consideration for the transport of live crabs that are often traded from European countries such as Ireland and Scotland to Asian regions such as the PRC and Hong Kong, where crabs may have to wait at ports to be tested [17,21]. However, it should be noted that Wiech, Frantzen, Duinker, Rasinger, and Maage [31] did not note an association between condition and cadmium levels.

The rates of uptake of cadmium in crustaceans appear to increase according to metal concentration, but are also determined by various other factors, including the uptake rates of other metals, such as zinc [79]. Indeed, it seems that even acute exposure to cadmium can result in an increase in its accumulation [80]. This implies that even a localised increase in cadmium levels in an area supporting the habitat of crabs could affect the levels of cadmium in the crabs, even over a short period of time. This is particularly concerning considering the long biological half-life of cadmium in the kidneys (10–30 years) [81]. Heavy industry can contribute to the geospatial occurrence of cadmium in fishing grounds, particularly around the mouths of some large European rivers [82]. Certainly, there is evidence of geospatial cadmium accumulation in crabs, as demonstrated by the higher cadmium levels in brown crabs in the north of Norway versus the south [31].

It is important to note that the crabs themselves may not always be unscathed by acute exposure. A study by Zhu et al. [83] determined that cadmium exposure induced the expression of stress-related genes and histological alterations in the gills and hepatopancreas of mud crabs (*Scylla paramamosain*). However, this was a laboratory experiment with cadmium levels ranging from 0 to 60 mg/L. Therefore, it is important to determine how relevant these experiments are to real-world situations. For instance, the study neglected to include another arm to examine the length of time required for the crab tissues to heal or return to homeostasis and to measure the internal cadmium uptake in relation to the pathology observed; however, in any case, their findings are a cause for concern. Another study found that 24 h of acute exposure to cadmium in Chinese mitten crabs (*Eriocheir japonica sinensis*) led to transcriptomic differences in the expression of genes relating to the immune and antioxidant defence functions of the crab [84]. These are not the only studies to investigate cadmium toxicity in crabs, as there is concern that cadmium may affect their reproductive capacity [85]. Likewise, a study in other crustaceans—crayfish (*Procambarus clarkii*)—indicated that cadmium may alter their gut histology and the function of their gut microbiota [86,87]. However, some species of crab may adopt mechanisms to mitigate cadmium toxicity. One study showed that the hepatopancreas of freshwater crabs (*Sinopotamon henanense*) altered the expression of a considerable amount of miRNAs in response to acute and subchronic cadmium exposure, which is thought to be an adaptive mechanism to prevent oxidative stress [88].

Considering that there may be histological and transcriptomic changes specific to cadmium exposure, there is the potential to develop rapid diagnostics to determine whether crabs have been exposed to cadmium. Although speculative, lateral flow tests akin to pregnancy tests in their operation could be designed for the detection of stress proteins related to cadmium exposure using haemolymph or another biological fluid. Such a test could be conducted on live crabs at sea, allowing for the release of the crab should an issue be detected. Further research is required to determine whether there are unique signatures in brown crabs that correlate with exposure to cadmium and could be leveraged to improve the industry. An alternative approach to mitigate cadmium levels in crabs is to alter the cooking process. One study has suggested that temperature and ultrasound could be used to reduce cadmium levels in crabs during the cooking process [89]. Such technology could be optimised to reduce cadmium levels in pre-packaged products such as cooked canned crab. Indeed, developing novel testing capacities or processing steps to reduce cadmium levels in crab products is necessary to save time and money and protect consumer health. Another potential way to reduce the risk of capturing cadmium-laden crabs is to carry out biomonitoring of other crabs or, indeed, other flora and fauna for cadmium bioaccumulation or related effects. For example, one could monitor the spermatozoa of *Mytilus galloprovincialis* for conformational alterations of protamine-like proteins, which have been shown to change as a result of cadmium exposure, potentially acting as early sentinels for the health of the environment [90]. However, this approach may be limited to crabs that are present close to the intertidal zone and may not be relevant to crabs that migrate far from the habitats of *Mytilus galloprovincialis*.

In summary, the sampling and analysis of cadmium in crabs is important, and there can be significant health and economic consequences if not properly conducted; hence, the necessity for standardised sampling, butchery, and analysis of cadmium in brown crabs. However, standardisation of protocols and even limits of cadmium in crabs is not consistent or uniform worldwide, even between trading nations.

## 5. Cadmium and Human Health

### 5.1. Cadmium Exposure in Humans

Understanding all possible routes of human cadmium exposure is important to consider when assessing one’s risk of excessive exposure. Cadmium exposure occurs through three possible routes: dermal, gastrointestinal, or pulmonary. Inhalation of cadmium by industrial workers or smokers is a significant exposure risk, but for the general population of non-smokers, exposure most commonly occurs via ingestion of contaminated foods or water [91]. Previously, it was suggested that atmospheric changes in cadmium levels due to increased pollution may affect blood cadmium levels. However, a recent study found that ingestion of dietary cadmium has a stronger impact on blood cadmium levels [92], likely due to the biomagnification of cadmium in dietary sources versus occasional acute exposure from atmospheric pollution. Therefore, it is important to consider dietary sources of cadmium, which may contain excessive cadmium, so that people and public health authorities can decide whether to mitigate excessive cadmium exposure risk by eliminating or reducing these food sources in the diet. Notably, cadmium is found ubiquitously in nature, and not all anthropogenic sources are the result of industrial emissions. For example, it has been documented that metal pollution can occur due to mining, aquaculture, wastewater treatment, crop farming, and animal breeding [93,94].

Diet is the most prevalent source of cadmium exposure in the general population, and it is also a source that can be mitigated to reduce cadmium exposure. It is estimated that daily dietary cadmium intake in unpolluted European areas can vary from 0.1 to 0.45 µg/kg bodyweight. However, in polluted areas, the total intake may be significantly more than the tolerable daily cadmium intake and reach several hundred µg/day [95,96]. This has major implications when considering the overall intake of cadmium from various foods, which tends to govern what limits are applied to the cadmium content of foods intended for human consumption. Lifestyle choices are certainly one of the biggest determinants of cadmium exposure. Smoking is a significant modifiable risk factor for cadmium exposure, as the tobacco plant accumulates cadmium from the soil into its leaves with great efficiency [97]. The United States national geometric mean blood cadmium level for non-smoking adults is 0.47 µg/L, whereas the mean of smokers is approximately thrice as high at 1.58 µg/L [98]. Smoking is estimated to at least double the body burden of cadmium exposure in one’s lifetime. Cadmium oxide (CdO) is a highly bioavailable form of cadmium that is responsible for the high concentrations of cadmium in the blood, urine, and tissues of smokers compared with non-smokers [99,100].

Whether there are specific foods that one should avoid, or dietary alterations required to reduce a person’s exposure to dietary cadmium is a topic of interest. The European Food Safety Authority (EFSA) noted that it is not the foods with the highest cadmium levels but, rather, the foods that are consumed in larger quantities most often that have the largest impact on dietary exposure to cadmium [1]. EFSA, using the food description and classification system FoodEx, determined that dietary cadmium exposure in European populations mainly originated from grains and grain-derived products (26.9%), vegetables and vegetable products (16.0%), and starchy roots and tubers (13.2%). In more detail, the following food categories contributed the most to dietary cadmium exposure across all age groups: potatoes (13.2%), bread and rolls (11.7%), fine bakery goods (5.1%), chocolate products (4.3%), leafy vegetables (3.9%), and molluscs (3.2%). However, it was noted that crustaceans were among a group of foods that exceeded 100 μg/kg, along with algal formulations, cocoa powder, offal, some seafood, mushrooms, and water molluscs [20]. Lifetime cadmium dietary exposure for Europeans is estimated to be approximately 2 μg/kg bodyweight/week (averaged for all age groups)—within the EFSA’s tolerable weekly intake (TWI) of 2.5 µg/kg bodyweight/week.

In Ireland, weekly adult intake of cadmium has been estimated to be between 1.1 and 2.5 µg/kg bodyweight/week, which is between 44 and 62% of the EFSA’s TWI [101]. These findings indicate that the majority of Irish people are not exposed to excess dietary cadmium levels. These findings are supported by the National Adult Nutrition Survey, which examined urinary cadmium excretion in the general population They and that 95% of participants had urinary cadmium levels below the 1 µg cadmium/g creatinine that the EFSA has deemed safe [102]. The main cadmium-contributing foods in the Irish diet were cereals (39%), vegetables (36%), and dairy (12%), where fish and shellfish only accounted for approximately 1% [101], likely due to the low consumption of fish and shellfish in Ireland.

**Figure 3:**
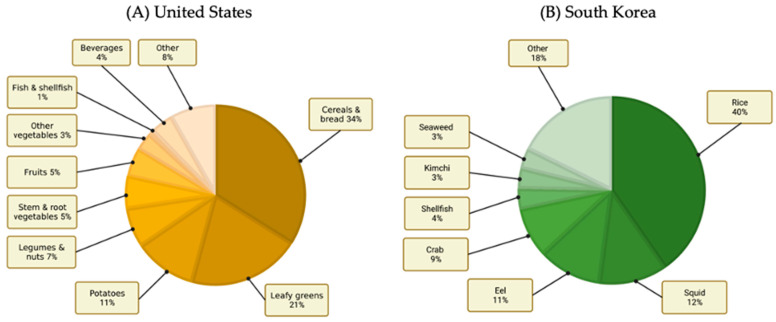
(**A**) The estimated contribution of major food components to cadmium intake as a percentage of total daily intake among a United States cohort of 12,523 aged 2 years and above in the NHANES 2007–2012 study, adapted from [103]. (**B**) The estimated percentage of the major food components contributing to daily cumulative cadmium intake in a South Korean cohort of 1245 people, adapted from [26].

In the United States, a recent study was conducted to determine the intake and sources of cadmium [103]. The average intake of dietary cadmium in the general population was 4.6 µg/day, or 0.54 µg/kg body weight/week—that is, approximately 22% of the tolerable weekly intake (TWI), which is considered to be 2.5 µg/kg body weight/week. However, certain demographics—such as elderly men, those who were well-educated and had a high income, and those with high adiposity—had higher levels of cadmium intake [103]. The food groups that contributed the most to the majority of the cadmium intake in the United States were cereals and bread (34%), leafy vegetables (20%), potatoes (11%), legumes and nuts (7%), and root vegetables (6%). Notably, the individual foods that contributed the most to the overall cadmium intake included lettuce (14%), spaghetti (8%), bread (7%), and potatoes (6%) (Figure 3A).

Interestingly, but unsurprisingly, due to the many cultures that coexist in the United States, there were ethnic and cultural differences in cadmium intake due to differences in dietary preferences. Lettuce was a major cadmium source for Caucasian and Black populations, whereas tortillas were the main source for Hispanics, and rice was the top contributor to the Asian population. Notably, the trends of cadmium intake in the United States seem to be very similar overall to those in the European Union. This is also unsurprising because despite there being many culinary differences in the foods and cultures of the US and Europe, the prevailing dietary pattern in both regions is the so-called “Western Diet” characterised by highly processed foods [104]. Amongst the Asian populations of the United States, smoking was the main exposure route for cadmium, followed by dietary exposure [105], which increases one’s risk for many non-communicable diseases—including cardiovascular, renal, and pulmonary diseases [104,106]. It is notable that fish and shellfish comprise a low contribution of cadmium to the American diet, but fish consumption is traditionally low in the United States [107].

Looking further afield, there are similarities between the so-called Western countries and Asian countries such as the PRC and South Korea. The average total daily cadmium intake in healthy Koreans is estimated to be 20.8 µg/day [26]. Figure 3B shows the food groups that contribute the most cadmium to the diet in South Korea. Notably, the food groups recorded are starkly different to the food groups associated with higher cadmium exposure in Europe or the United States. In particular, they seem to be culturally relevant. For example, there are much higher levels of rice (40.3%), as was noted in Asian groups from the United States cohort [103], but also higher intake of seafood and specific foods associated with Korean cuisine, such as kimchi and seaweed. Indeed, crab in this case was shown to contribute 8.6% of the cumulative cadmium intake in this South Korean population.

Many parts of the PRC share similar food consumption patterns to South Korea and, thus, similar cadmium exposure [108]. In one study [108], freshwater crab and sea-caught crab samples obtained contained 0.101 ± 0.323 and 0.544 ± 1.203 mg/kg (mean ± standard deviation) cadmium, respectively, which were estimated to be consumed at 0.7 ± 7.3 and 0.8 ± 8.9 g/day by the general population. This contrasts with rice and wheat, which contain 0.062 ± 0.128 and 0.021 ± 0.026 mg/kg of cadmium and are consumed at 218 ± 174.5 and 145.4 ± 168 g/day (mean ± standard deviation), respectively. These data show that while it is true that crab contains higher concentrations of cadmium in mg/kg weight, its consumption levels are markedly different. A further examination of the specific food groups contributing dietary cadmium to the Chinese population is presented in Table 2. The interesting point of this research is that for the high-exposure subpopulation with cadmium exposure higher than the 95th percentile, rice was the largest contributor (58.6%), followed by shellfish (13.2%), and leafy vegetables (9.2%). This is a very small sub-fraction of the population that are exposed to such high levels of cadmium because of their dietary choices; it would be interesting to break down the subcategory of shellfish further to determine the impact that crab may have on the consumption of cadmium in this cohort. The study determined that the mean dietary cadmium exposure of the general Chinese population was 15.3 μg/kg body weight/month (30.6 μg/day for a 60 kg average body weight of adults). A similar study in Shanghai found that the average exposure to dietary and environmental cadmium was 167 µg/day (34% of the PTDI). Similarly, vegetables and rice were the main sources of dietary cadmium, and tobacco accounted for 25% of the total cadmium exposure from non-occupational sources [109]. Considering that almost 20% of agricultural soil in the PRC is contaminated with cadmium [110], it is likely that their dietary exposure to cadmium will only increase with their growing economy. Furthermore, the daily exposure to dietary cadmium in the Chinese population is significantly higher than that in either Europe or the United States.

The total diet study (TDS) is a food safety monitoring program that is conducted by various food agencies, including the United States Food and Drug Administration (FDA), the Food Standards of Australia and New Zealand (FSANZ), and the European Food Safety Agency (EFSA) [111]. These are “market basket surveys” that collection of various food samples from groceries and retailers for the quantitation of food additives, pesticide residues, contaminants, nutrients and, of course, heavy metals [112,113]. The TDS provides a realistic approach to gauge the relative contribution of each food group and specific item to estimate the total intake of cadmium in the diet. Foods that were consumed in large quantities at high frequency contributed the most to cadmium intake [111]. Currently, TDS data are available for a limited number of countries, including Australia, the United States, France, Spain, Sweden, Chile, Denmark, and Serbia [100]. Overall, data from TDS show that these countries’ cadmium intake varies between 8 and 25 µg/day for the average consumer with staple foods (e.g., rice, wheat, and potatoes), which accounted for 40–60% of total dietary cadmium ingestion. Shellfish, crustaceans, molluscs, offal, and spinach were considered to be additional cadmium sources [100,111]. These types of studies are often thought to underestimate dietary cadmium, as they fail to demonstrate an association between estimated cadmium intake and the incidence of cancer and bone diseases [114,115,116,117].

Overall, these epidemiological and dietary studies demonstrate that dietary cadmium exposure is affected by many factors and that the main contributor of dietary cadmium in all instances around the world mostly originates from staple foods such as rice, wheat, and other grains. In Asian diets, seafood and shellfish were contributors to dietary cadmium intake, but this is not necessarily the main source in countries that consume Western diets. Overall, it seems that it is important to strike a balance and be cautious of foods that are potentially significant contributors of cadmium to the diet. Indeed, moderate consumption of shellfish should not significantly affect one’s risk of illness from cadmium ingestion, but further research specific to crab consumption is required.

### 5.2. Cadmium Ingestion and Accumulation in Humans

Depending on the exact dose and nutritional composition of a food, the human gastrointestinal tract can take up 3–5% of ingested cadmium [20,118]. Various factors can affect cadmium uptake in humans, such as low intakes of calcium, vitamin D, zinc, and copper [91]. One possible mechanism of high cadmium resorption is related to the assumption that cadmium shares molecular homology with zinc and calcium; as a result, low levels of these minerals are compensated by higher cadmium resorption [119]. This observation was closely replicated in competitive resorption studies in rats against other polyvalent cations such as Cr^3+^, Mg, Ni, Pb, and Sr [120]. Notably, a low zinc/iron status in individuals who subsist on diets characterised by high rice intake may cause high absorption of cadmium in contrast to other staple diets [121]. Other factors that affect cadmium uptake include gender, nutritional status, diet, and smoking status can also affect the bioavailability of cadmium in humans [68].

Indeed, various human studies show that cadmium intake can be increased by dietary fibre intake [96]. Animal experiments have shown that diets with high concentrations of protein and lipids can also increase net intestinal uptake of cadmium and that diets high in wheat bran may reduce cadmium intake [95]. The exact mechanisms of these effects on cadmium intake are yet to be fully elucidated. On the other hand, cadmium can bind to low-molecular-weight proteins rich in cysteine such as metallothionein, which may increase its bioavailability [122]. This has been demonstrated naturally in various marine organisms where cadmium seems to be bound to small, soluble cytoplasmic proteins, including in oysters, mussels, scallops [122], and green crab (*Carcinus maenas*) [62,63,123]. In rat studies, cadmium binds to amino acids and peptides in the intestinal tract [124], which undoubtedly has implications for its bioavailability. What these studies suggest is that these effects may be the result of a food matrix effect in a similar way to dairy products, where nutrients are more or less bioavailable depending on the food’s structure and composition [125]. This implies that the foods or ingredients that we mix with foods containing high cadmium levels may affect the overall bioavailability of cadmium. Therefore, it may be possible to mitigate cadmium’s bioavailability when preparing foods that may have higher levels of cadmium by altering the food matrix. However, research is very limited in this area, and further studies are required to confirm such associations.

Evidence from animal studies shows that marginal deficiencies in zinc, iron, and calcium can enhance the absorption, organ accumulation, and retention of dietary cadmium [121]. Moreover, marginal deficiencies can enhance cadmium absorption as much as 10-fold in diets containing low cadmium concentrations similar to those consumed by some human populations, indicating that people who are nutritionally marginal with respect to zinc, iron, and calcium are at higher risk of cadmium-related diseases than those who are nutritionally adequate [126,127,128]. Indeed, similar studies in humans show that an individual’s iron levels may be a metabolic factor of concern in the resorption of cadmium. It has been demonstrated that a lack of iron leads to a 6% higher uptake of cadmium in individuals with normal iron levels [129]. A study of iron-deficient children in the United States found elevated blood cadmium levels [130]. This accounts for higher cadmium absorption in individuals with a habitual iron deficit (e.g., children or menstruating women) or people with anaemia [91]. It seems that these observations are the result of the expression of DCT-1 and MTP1—metal ion transporters in the gastrointestinal tract that act as a gate for cadmium resorption when low iron levels occur [131,132]. Overall, the evidence presented supports the notion that dietary components and trace element status can affect the fractional intestinal uptake of cadmium, as reviewed by Andersen et al. [95].

These findings hint at the possibility of ensuring that individuals who may be at high risk of exposure to cadmium have a healthy nutritional status. Those who may be deficient in some minerals may consider dietary alterations or dietary supplements to ameliorate mineral deficiencies.

### 5.3. Cadmium’s Transport, Bioavailability, and Excretion in Humans

Cadmium is well-known for its toxicity to humans, as evidenced by decades of observational studies and research. Like many heavy metals, bioaccumulation of cadmium in mammals can differentially affect certain tissues, including bone, the liver, muscle, and the kidneys. Indeed, Cd^2+^ is dangerous in that it can substitute for Zn in enzyme structures. Likewise, calcium and cadmium have similar ionic radii (109 pm and 114 pm, respectively), meaning that cadmium can accumulate in the bone along with calcium [133].

Once taken up by the gastrointestinal tract and deposited into the bloodstream, cadmium binds to proteins such as albumin and metallothionein. From there, it is transported to the liver, where cadmium can induce the production of metallothionein. Following the necrosis and apoptosis of hepatocytes, cadmium–metallothionein (Cd–M) complexes form, which are washed from sinusoidal blood. Some cadmium then enters the enterohepatic cycle via secretion into the biliary tract in the form of cadmium–glutathione conjugates. Cadmium can then be enzymatically degraded to cadmium–cysteine complexes in the biliary tree, where it can re-enter the small intestine [91,134]. Cadmium accumulates in the renal tubular cells in the cortex of the kidneys via the transport of metallothionein. It resides there, where it can have a half-life of 10–30 years [135]. Lifelong exposure to and consumption of foods containing cadmium can lead to the accumulation of cadmium, and as it is very slowly excreted from the body, it causes irreversible tubular cell necrosis in the kidneys [91]. Unfortunately, the kidneys are the organs most susceptible to damage from cadmium accumulation [136], although chronic and prolonged exposure to cadmium can have devastating effects on various tissues of the human body and can even cause bone demineralisation [137]. When cadmium arrives at the kidneys in the form of Cd–M, it is filtered in the glomerulus and reabsorbed in the proximal convoluted tubules, where it tends to remain [91].

Cadmium concentrations can be measured in urine, hair, blood, nail, and saliva samples. Cadmium-induced kidney damage correlates with urinary cadmium excretion. Indeed, proteinuria characterised by the excretion of low-molecular-weight proteins such as retinol-binding protein or ß_2_-microglobulin [138] is likely to occur with a 10% response rate when the concentration of cadmium in the cortex exceeds approximately 200 μg/g wet weight (200 ppm) [135]. Moreover, urinary cadmium has been used as a non-invasive detection method of the accumulation of cadmium in the kidneys, and as a marker of tubular dysfunction in industrial workers and those who have had low environmental exposure. This is due to the curvilinear relationship between urinary cadmium and cadmium accumulation in the kidneys [118,139]. This allows for the urinary cadmium value corresponding to the critical kidney cadmium level of 200 ppm to be estimated at 10 μg/g creatinine, which is estimated in concordance with the relationship between urinary cadmium and proteinuria [138,140,141]. These measurements are now well-established and, in populations with excessive exposure to cadmium, urinary cadmium is correlated with the renal cadmium levels or body burden. Worryingly, these levels remain elevated many years after cessation of exposure [142].

While measuring ß_2_-microglobulin was previously thought to be the most reliable and accepted method of measuring cadmium burden and levels in humans, there are several other urinary biomarkers for the assessment of the renal effects of cadmium. A significant debate about the utility of these various biomarkers is ongoing [111]. These markers are outlined in Table 3 as per the publication of Satarug [111]. The associated renal biological effects are also enclosed in Table 3. These biomarkers are currently being used to assess the impacts of seafood and crab consumption on human health [143,144].

An interesting in vivo study assessed the bioavailability of cadmium from boiled crab hepatopancreas, inorganic cadmium, or dried wild mushroom fed to mice [145]. The study design included a control group of mice that received low levels of cadmium (<0.007 ppm) in their feed, which did not lead to detectable levels of cadmium over a 9-week exposure period. The authors used cadmium accumulation in the kidneys and liver as a measure of absorption. Notably, the bioavailability of cadmium from boiled crab hepatopancreas was lower than that of cadmium from mushroom or even inorganic cadmium. Cadmium in the crab hepatopancreas is mainly associated with denatured proteins with low solubility, whereas a large proportion of cadmium in dried mushroom is associated with soluble ligands. Therefore, there was an indication that the difference in cadmium speciation might account for the lower bioavailability of cadmium from crab than from mushroom. However, the authors commented that the difference in bioavailability was low, and that restricting intake was recommended if the products were high in cadmium. This may be evidence of cadmium speciation or, indeed, a food matrix effect. A similar study in which rats consumed a diet consisting of high crab intake (4 mg/kg organic-bound cadmium), a low-crab diet (0.2 mg/kg organic-bound cadmium), or a casein-based cadmium diet (4 mg/kg as cadmium chloride) for 6 months showed that cadmium intake from the high-crab diet was only half that of the diet consisting of cadmium chloride [146]. These findings also appear to indicate that there may be a food matrix effect at play. Other studies in humans have shown that cadmium is more bioaccessible from fish (84%) than from shellfish (73%) [147]. Worryingly, individuals who smoke cigarettes and have a high consumption of seafood can experience exacerbated adverse effects of cadmium exposure [147]. This is particularly dangerous for populations such as the PRC, where many of the people smoke frequently. There are still many questions regarding cadmium’s bioavailability that require further investigation, particularly regarding the food matrix effect and how it may be leveraged to mitigate dietary cadmium intake.

Another point to note is that current health risk assessments relating to cadmium exposure in humans rely heavily on the evaluation of the toxicity to the kidneys alone. In 2010, the Joint Food and Agriculture Organisation (FAO) and World Health Organisation (WHO) Expert Committee on Food Additives and Contaminants (JECFA) deemed the kidneys to be a suitable target for evaluating cadmium toxicity, as measurements of ß_2_-microglobulin could be used as a surrogate biomarker for the effects of dietary cadmium intake [148]. The JECFA established a tolerable monthly intake of 25 µg/kg/bodyweight per month, with a urinary cadmium excretion rate of 5.24 µg/g creatinine or 0.8 µg/kg/day as a nephrotoxicity threshold [148,149,150]. While the EFSA and JECFA share the same critical ß_2_-microglobulin endpoint of 300 µg/g creatinine, the EFSA adopted a different cadmium excretion rate of 1 µg/g creatinine as the nephrotoxicity threshold, along with an uncertainty factor of 0.36 µg/kg bodyweight per day for 50 years as a benchmark dose [151]. While these values are important references to monitor to stay within safe levels of cadmium exposure, relying on one biomarker (ß_2_-microglobulin) is insufficient. In 2019, Satarug et al. [152] showed that ß_2_-microglobulin excretion levels as low as 100–299 µg/g creatinine were associated with a 4.7-fold increase in eGFR to ≤60 mL/min/1.73 m^2^—a measurement consistent with chronic kidney disease. Therefore, a ß_2_-microglobulin endpoint of 300 µg/g creatinine may not be a low enough threshold to detect early nephrotoxicity [149].

Considering the emerging evidence that many organ systems are affected by cadmium exposure, other toxicity endpoints may be informative for risk assessment. As reviewed by Satarug et al. [149], other biomarkers of chronic low-dose cadmium exposure may contribute to risk assessments. For example, reductions in estimated glomerular filtration rate (eGFR) and lower fecundity have been observed at cadmium excretion levels as low as 0.5 µg/g creatinine, with worsening outcomes noted in a dose-dependent manner [149]. In men, sperm cadmium levels are inversely associated with sperm motility [153,154] and appear to be associated with other measures of sperm quality, viability, and acrosome reactions [149]. In females, high blood cadmium levels have been associated with infertility [155]. High urinary cadmium levels (~0.70 µg/L) have been associated with ovarian reserve depletion and ovarian insufficiency, with serum follicle-stimulating hormone (FSH) levels ≥ 10 IU/L [156] and ≥25 IU/L [157], respectively. However, sampling and monitoring of reproductive health is intrusive and inconvenient; therefore, surrogate markers such as serum FSH or anti-Mullerian hormone (AMH) in females may be useful. Blood biomarkers are preferred because of the convenience of analysing a blood sample. Therefore, alternative approaches have been sought, including monitoring of epigenetic factors [158]. Preliminary research indicates that cadmium exposure induces epigenetic changes in micro ribonucleic acids (miRNAs) that may lead to the development of novel blood-borne biomarkers [159,160]. Collectively, these findings indicate that additional novel biomarkers of human cadmium exposure are necessary to determine one’s risk of toxicity and disease, as opposed to the reliance on monitoring kidney function alone.

### 5.4. Cadmium Toxicity in Humans

Cadmium can affect important cellular functions such as cell differentiation, proliferation, and apoptosis, which is of concern considering that these processes overlap with the important processes of the generation of reactive oxygen species (ROS) and DNA repair mechanisms [81]. Cadmium at low concentrations even has the capacity to bind to mitochondria and can inhibit cellular oxidative phosphorylation and cellular respiration [161]. Cadmium exposure results in chromosomal aberrations, DNA strand breaks, sister chromatid exchange, and DNA–protein crosslinks. Cadmium can potentially cause mutations and chromosomal deletions [162]. Cadmium toxicity encompasses the depletion of reduced glutathione (GSH), binds sulfhydryl groups with proteins, and causes the enhanced production of ROS, resulting in oxidative stress, which may promote organ toxicity, apoptotic cell death, and carcinogenicity [81]. Cadmium can also inhibit the capacity of the natural antioxidant enzymes, such as catalase, manganese superoxide dismutase, and copper/zinc-dismutase [163]. Metallothionein is also involved in these processes and can act as a free-radical scavenger of hydroxyl and superoxide radicals [164]. Largely, the cells that contain metallothioneins are resilient to the effects of cadmium toxicity. However, it has been observed that cells that do not synthesise metallothioneins are sensitive to cadmium [81].

Cadmium has also been shown to be an endocrine disruptor. Cadmium may affect thyroid function, as demonstrated in both animal and human studies [165], where tissue damage in the thyroid led to hyperplasia and hypertrophy [166,167,168]. Moreover, cadmium has been linked with changes in hormone function [169,170], and there is suspicion that chronic cadmium exposure may lead to thyroid cancer, but further research is required [171]. Cadmium may also act as a metalloestrogen, as it can bind to the oestrogen receptor [172], which has led to a concern that chronic cadmium exposure may be associated with breast cancer [173,174,175]. There have also been links drawn between cadmium and the inhibition of progesterone synthesis, ovarian and reproductive tract morphological alterations, disruption to menstrual cycles, and issues with pregnancy and birth [176]. Likewise, cadmium may mimic some of the effects of androgens and may play a role in prostate cancer [177,178] and reduce male fertility by affecting spermatogenesis and motility [179].

**Figure 4:**
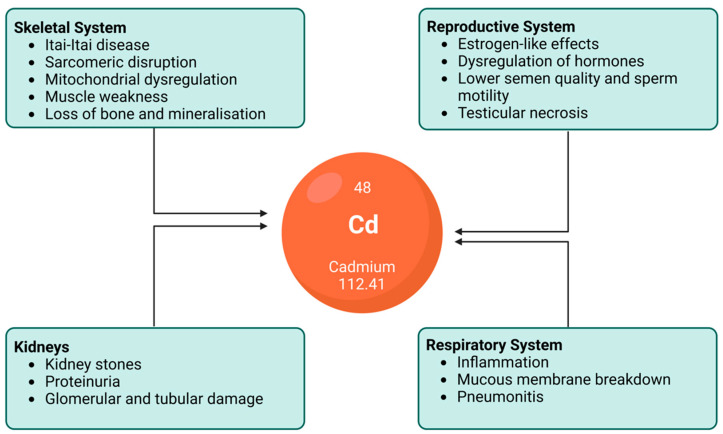
An illustration depicting the effects of chronic cadmium exposure on various human organs and systems. While not an exclusive list, this figure demonstrates the importance of mitigating excess cadmium ingestion.

The vast and various effects of cadmium exposure on the human body that have been explored in the previous sections lead to various clinical manifestations. As such, it is known that different forms of cadmium compounds lead to different clinical manifestations. However, the details of this require further investigation. While cadmium poisoning is very rare, it can happen. Itai-itai disease is the most severe form of chronic cadmium toxicity in humans, caused by the prolonged ingestion of cadmium. Areas severely polluted by cadmium, such as the Jinzu River Basin in Toyama, Japan, have high incidences of cadmium-related pathologies. In that example, the river was polluted with slag from a mine upstream. The cadmium-polluted water was subsequently used to irrigate crops and rice between the 1910s and 1960s. The water from this river was used as potable water and for cooking, bathing, etc. [25]. This was significant, as cadmium is a food-chain contaminant that has high rates of soil-to-plant transference [111] and, thus, a high risk of ingestion. Itai-itai disease is characterised by renal tubular disorder and renal osteomalacia [180]. Even if people did not get itai-itai disease in the Jinzu Basin, they were at serious risk of cancer [181]. Some of the main effects of cadmium on the human body are presented in Figure 4. Patients with cadmium toxicity require significant treatment, including gastrointestinal tract irrigation, supportive care, and chemical decontamination via traditional chelation therapy with novel chelating agents and nanoparticle-based antidotes [81].

## 6. Crab Consumption, Cadmium, and Human Health

There are limited studies that have investigated the effects of crab consumption on cadmium ingestion and human health. A recent study examined whether regular crab meat consumers exhibited increased levels of ß_2_-microglobulin or cadmium in their urine compared to those who did not eat crab meat [143]. They determined that whole blood cadmium levels can be both a short- and long-term marker of cadmium intake. However, while it was expected that cadmium levels would be elevated in the crab meat consumers, the study showed that crab meat consumers did not show increased levels of urinary cadmium and, consistent with this, showed no changes in cadmium-induced kidney toxicity markers. Consequently, the authors concluded that compared to consumers who reported very little crab meat consumption, healthy middle-aged consumers who regularly consumed brown crab meat products (an average of 447 g/week) for an average of 16 years showed no changes in long-term cadmium exposure or kidney toxicity. A study of French seafood consumers demonstrated that the mean dietary ingestion of cadmium was 2.4 ± 3.3 mg/kg bodyweight/week. The authors also determined that the mean urinary cadmium level was 0.65 ± 0.45 mg/g creatinine, and was significantly higher in women than in men. This is particularly interesting, as sexual dimorphism was observed in populations of Japan who suffered itai-itai disease, where women generally had a more severe prognosis [100].

In the United States, the Long Island Study of Seafood Consumption, conducted in New York, examined the relationship between seafood intake and blood cadmium levels in 252 people who were avid seafood consumers [182]. After the researchers adjusted for age, BMI, sex, smoking status, and other factors, a linear regression model was employed. They determined that there was no association with regular seafood intake (β = −0.01; *p* = 0.11) but did identify an association between salmon intake in cups/week (ln transformed) (β = 0.20; *p* = 0.001) and blood cadmium levels. The study determined that only salmon was meaningfully associated with blood cadmium levels and that seafood was most likely not a significant source of cadmium exposure. They suggested that as the cadmium levels in salmon are not higher than those in many other seafood species, the association with salmon intake was likely due to higher consumption of salmon within this cohort. A similar study—the Norwegian Fish and Game study—assessed cadmium concentrations in spot urine and blood samples and conducted a food frequency questionnaire (FFQ) with 179 volunteers. The median urinary cadmium level was 0.16 μg/L when corrected for creatinine, and the median (5th to 95th percentile) blood cadmium level was 0.45 μg/L. According to the FFQ, 24% of individuals designated as high cadmium consumers and 8% of the controls (i.e., those with lower levels of cadmium in the diet) had intakes above the TWI. Notably, there was an association between high cadmium levels and seafood consumption, which was thought to be partially driven by crab consumption [183]. This may be unsurprising, as both white and brown crab meat is consumed in Norway and is a contributor of cadmium to the diets of Norwegian seafood consumers [28].

Considering these collective findings and some of the mechanisms of cadmium’s bioavailability discussed in this review, it is likely that while it is necessary to monitor cadmium levels in foods, these levels may not directly translate to 1:1 absorption from the bioavailable cadmium pool. It is likely that the food matrix also plays a considerable role in cadmium’s bioavailability. As discussed previously, the preparation of crab may also play a significant role in the cadmium levels of crab for consumption [30]. Finally, it is likely that the health and nutritional status of the individual consuming the product, along with various other factors, contribute to whether a person is at risk of cadmium bioaccumulation and associated negative health effects.

## 7. Conclusions

Trace cadmium is naturally present in the food chain due to its ubiquitous presence in nature. Some food sources, including crustaceans such as the brown crab, naturally bioaccumulate cadmium and, therefore, are thought to pose a health risk. However, cadmium exposure from dietary sources may be mitigated by individuals and public health authorities by limiting exposure. As discussed in this review, evidence supports the notion that moderate consumption of brown crab is unlikely to pose a significant health risk when one’s lifestyle and dietary choices offer little risk of excessive cadmium exposure. In particular, evidence supports the safe consumption of white crab meat due to its low cadmium levels and other beneficial health benefits, including its role as a source of protein and omega-3 fatty acids. On the other hand, the brown meat containing the hepatopancreas does have high levels of cadmium, but this is unlikely to pose a significant health threat if the brown meat is consumed in low amounts. However, regular consumption of the brown meat is not recommended until further dietary research deems frequent consumption safe. Finally, as discussed, there are discrepancies and various interpretations of adequate testing for cadmium in crab products in the industry. Furthermore, the sampling, butchery, and analysis of brown crab would appear to vary from region to region. Differences in legislation and interpretation of cadmium’s risks have led to rifts in the export trade of live crabs between Europe and Asia, which have caused significant issues to trade for exporters such as Ireland and the United Kingdom. This review also raises questions regarding how legislation is put forward and what are the most reasonable assessments to make when considering individual and public health risks. Collective agreement on how to determine cadmium risk factors and the standardisation of crab monitoring are required to ensure a safe and equitable crab market internationally.

## Figures and Tables

**Table 1:** An overview of cadmium concentrations in brown crab (*Cancer pagurus*) expressed as mg/kg ww.

Crab Meat Type (State)	Location	Estimated Cadmium Levels (mg/kg)		Detection Method	Reference
		Mean ± SD			
		Spring Caught	Summer Caught		
White meat (raw)	Portugal	0.07 ± 0.06	0.01 ± 0.01	FAAS	[14]
White meat (steamed)	Portugal	0.24 ± 0.38	0.10 ± 0.14		
White meat (boiled)	Portugal	0.05 ± 0.05	0.10 ± 0.16		
Brown meat (raw)	Portugal	8.4 ± 8.3	8.1 ± 14.2		
Brown meat (steamed)	Portugal	7.6 ± 5.2	11 ± 13		
Brown meat (boiled)	Portugal	5.6 ± 5.6	5.0 ± 8.2		
		Mean ± SD			
White claw meat (raw)	Northern Norway	0.024 ± 0.012		ICP-MS	[30]
White claw meat (raw)	Southern Norway	0.007 ± 0.005			
Brown meat (raw)	Northern Norway	1.15 ± 0.76			
Brown meat (raw)	Southern Norway	0.21 ± 0.14			
White claw meat (boiled)	Northern Norway	0.30 ± 0.29			
White claw meat (boiled)	Southern Norway	0.065 ± 0.075			
Brown meat (boiled)	Northern Norway	0.45 ± 0.26			
Brown meat (boiled)	Southern Norway	0.16 ± 0.12			
		Yearly median concentration range between 2016 and 2017			
Brown meat (raw)	Mausund, Norway	2.11–4.37		ICP-MS	[33]
		Estimated mean			
White meat (raw)	English Channel	0.10		FAAS	[13]
Brown meat (raw)	English Channel	15–18			
White meat (raw)	Scottish coast	0.10			
Brown meat (raw)	Scottish coast	20–30			
		Mean/range			
White meat (raw)	Birsay, Scotland	-/0.08–0.27		FAAS	[64]
Brown meat (raw)	Birsay, Scotland	7.30/1.12–49.4			
White meat (raw)	Norwegian coast	0.62/0.002–4.5		ICP-MS	[33,65]
Brown meat (raw)	Norwegian coast	8.7/0.24–43.0			
White meat (raw)	Senja, Norway	0.53/0.03–3.2		ICP-MS	[33,66]
Brown meat (raw)	Senja, Norway	9.3/1.6–29.0			
White meat (raw)	Kvaløya, Norway	0.25/0.06–0.74			
Brown meat (raw)	Kvaløya, Norway	30.0/7.3–58.0			

**Table 2:** Data depicting the main contributors to dietary cadmium intake in (A) the general Chinese population and (B) the highly exposed Chinese population. Data adapted with permission from [108].

General Population (A)		High-Exposure Population (B) *	
Food Group	Percentage (%) Contribution of Dietary Cadmium Intake	Food Group	Percentage (%) Contribution of Dietary Cadmium Intake
Rice	55.8	Rice	58.6
Leafy vegetables	10.5	Leafy vegetables	9.2
Wheat flour	11.8	Wheat flour	2
Shellfish	4.8	Shellfish	13.2
Meat	2.6	Meat	2
Seaweed	2.4	Seaweed	6.4
Other vegetables	2.4	Other vegetables	1.4
Other cereals	2.1	Other cereals	0.9
Root and stalk vegetables	2.0	Root and stalk vegetables	1.7
Mushrooms	1.1	Mushrooms	1.5
Fish	1.1	Fish	1
Legumes	0.9	Legumes	0.6
Fruits	0.6	Fruits	0.4
Eggs	0.6	Eggs	0.2
Nuts	0.4	Nuts	0.4
Offal	0.4	Offal	0.2
Other	0.5	Other	0.3

* The highly exposed population was determined to be those within the 95th percentile of the mean dietary cadmium exposure of the general Chinese population.

**Table 3:** Urinary biomarkers for the assessment of cadmium burden on the kidneys. Adapted from Satarug [111].

Biomarkers	Abnormal Values	Interpretations and Associations
NAG	>4 U/g creatinine	Tubular injury, mortality
Lysozyme	>4 mg/g creatinine	Tubular injury
Total protein	>100 mg/g creatinine	Glomerular dysfunction, CKD
Albumin	>30 mg/g creatinine	Glomerular dysfunction, CKD
ß_2_MG	≥1000 µg/g creatinine	Irreversible tubular dysfunction
ß_2_-MG	≥300 µg/g creatinine	Mild tubular dysfunction, rapid GFR decline
ß_2_-MG	≥145 µg/g creatinine	Increased hypertension risk
α1-MG	≥400 µg/g creatinine	Mild tubular dysfunction
α1-MG	≥1500 µg/g creatinine	Irreversible tubular dysfunction
KIM-1	≥1.6 mg/g creatinine in men ≥2.4 mg/g creatinine in women	Kidney injury, urinary KIM-1 levels correlated with blood cadmium levels

Abbreviations: NAG = N-acetyl-β-D-glucosaminidinase; ß_2_-MG = beta-2 microglobulin; α1-MG = α1-microglobulin; KIM-1 = kidney injury molecule-1; CKD = chronic kidney disease; GFR = glomerular filtration rate.
